# Dr. Upendranath Brahmachari: The Unsung Hero of Indian Medical Research

**DOI:** 10.7759/cureus.66148

**Published:** 2024-08-04

**Authors:** Umesh Kawalkar, Amar Mankar, Abhay Gaidhane, Manoj Talapalliwar

**Affiliations:** 1 Community Medicine, Government Medical College, Akola, Akola, IND; 2 Community Medicine, Datta Meghe Institute of Higher Education and Research, Wardha, IND; 3 School of Epidemiology and Public Health, Jawaharlal Nehru Medical College, Datta Meghe Institute of Higher Education and Research, Wardha, IND

**Keywords:** nomination for nobel prize, knighthood, historical vignette, dermal leishmanoid, urea stibamine, kala-azar

## Abstract

Upendranath Brahmachari (1873-1946) was a prominent Indian scientist and physician renowned for his groundbreaking work in tropical medicine. He is most famous for discovering urea stibamine, a highly effective treatment for kala-azar (visceral leishmaniasis), a deadly parasitic disease. This discovery had a significant impact on public health, saving countless lives in India and beyond. Born in Jamalpur, Bihar, Brahmachari pursued medical education at the University of Calcutta, where he later became a professor. His dedication to medical science earned him numerous accolades, including a knighthood in 1934. In 1929, Brahmachari was nominated for the Nobel Prize in Physiology or Medicine in recognition of his work on urea stibamine. Although he did not win, the nomination underscored the global significance of his contributions. In addition to his scientific achievements, Brahmachari was active in public service, advocating for improved healthcare and medical education in India. His legacy continues to inspire medical professionals and researchers worldwide.

## Introduction and background

Early life and education

**Figure 1:**
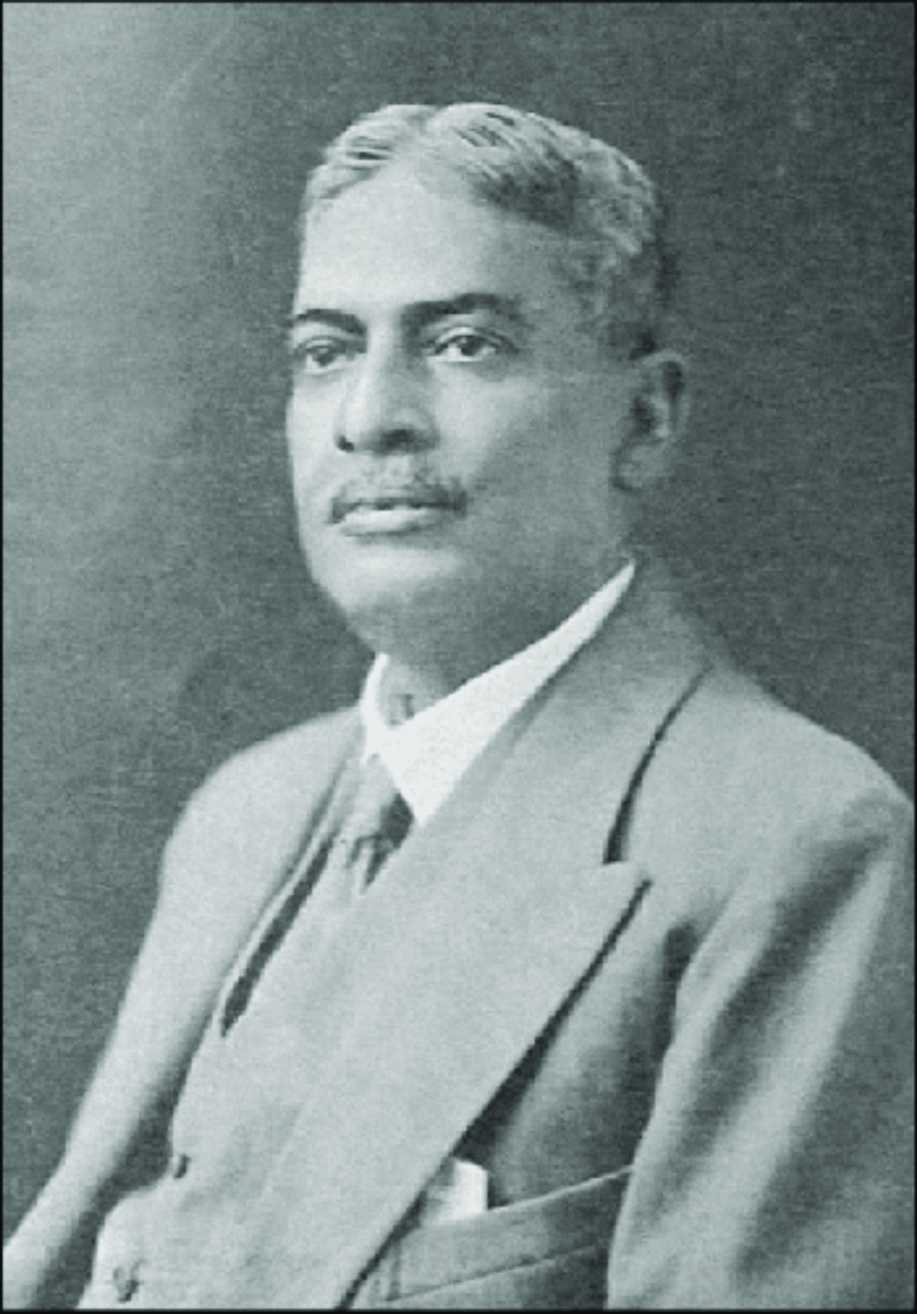
Dr. Upendranath Brahmachari. Image courtesy of Wikimedia Commons (public domain).

Dr. Upendranath Brahmachari (Figure 1) was born on December 19, 1873, in Sardanga village near Purbasthali, District Burdwan, West Bengal, India. His father, Dr. Nilmani Brahmachari, was a respected physician working with the Eastern Railways, and his mother, Saurabh Sundari Devi, was a homemaker. Upendra studied at the Eastern Railways Boys’ School in Jamalpur, where he displayed a keen intellect and passion for learning. In 1893, he graduated with a BA degree from Hooghly Mohsin College, securing double honors in Mathematics and Chemistry and winning the Thwaites medal for excelling in Mathematics. The following year, Brahmachari earned his master’s degree in Chemistry from Presidency College, Kolkata. Despite his proficiency in Chemistry, he shifted his focus to Medicine and joined Calcutta Medical College. He excelled in his medical studies, completing his Licentiate in Medicine and Surgery in 1899 and achieving the highest marks in both Medicine and Surgery, for which he received the Goodeve and McLeod medals [1]. In 1902, Brahmachari earned his MD degree from Presidency General Hospital in Calcutta. He was assigned the position of a teacher for Physiology and Materia Medica at Dacca Medical School, where he worked under Sir G. Bomford. His research during this period focused on hemolysis, culminating in a PhD in 1904 with his thesis titled “Studies in Haemolysis.” In 1905, Brahmachari returned to Calcutta and joined Campbell Medical School (now Nil Ratan Sircar Medical College and Hospital) as a Teacher of Medicine and First Physician. He dedicated approximately 20 years to teaching and clinical practice at this institution. In 1923, he joined Calcutta Medical College, where he continued to teach until his retirement in 1927. Even after retirement, he remained active in the medical field, serving as a professor specializing in Tropical Diseases at Carmichael Medical College and taking charge of the Tropical Disease Ward at the National Medical Institute. He also served as the Head of the Department and Honorary Professor of Biochemistry at the University Colleges of Science [1,2].

## Review

Dr. U.N. Brahmachari’s groundbreaking research on kala-azar

In 1824, the first reports of a mysterious and lethal epidemic emerged from Jessore district, now part of Bangladesh. This epidemic, which would later be known as “Burdwan fever,” resurfaced in Burdwan district, Lower Bengal, in 1860, causing widespread mortality and impacting agriculture and government revenues. By 1870, Charles J. Jackson, the Sanitary Commissioner of Bengal, documented the severe impact of the outbreak around Burdwan. In the decades that followed, reports of a similar fever began to surface from the Garo Hills in Assam, leading Sir Leonard Rogers to identify the disease as kala-azar, or visceral leishmaniasis [2]. The disease continued to spread throughout the Gangetic Plain and the Brahmaputra Valley, leading to a considerable decrease in population between 1891 and 1901. Ronald Ross, commissioned in 1898 to investigate kala-azar, initially concluded it was a form of degenerated malaria, but this theory did not fully resolve the nature of the disease. Meanwhile, in the early 1900s, William Leishman, working at the Royal Victoria Hospital at Netley, identified unknown parasites in the spleen of a soldier who had died near Calcutta. Leishman speculated that the disease might be a form of trypanosomiasis. Around the same time, Charles Donovan at Madras Medical College made similar observations and corresponded with Ross, who named the parasites Leishman-Donovan bodies. The parasite was later classified as *Leishmania donovani*. The initial treatment breakthrough came from Alphonse Laveran, who demonstrated that arsenic-based compounds could kill trypanosomes. By 1912, Gasper de Oliviera Vianna in Brazil had successfully used tartar emetic (potassium antimonyl tartrate) to treat skin lesions caused by *Leishmania braziliensis*. These successes led doctors in India, including Leonard Rogers, to begin using tartar emetic for kala-azar treatment by 1915 [3,4].

However, the treatment was highly toxic and required a lengthy regimen, making it challenging for patients to complete. Brahmachari sought to develop a safer and more efficient alternative. Owing to the success of atoxyl, an organic arsenic compound used to treat sleeping sickness, he began experimenting with organic antimonials. After years of diligent research and experimentation, often under challenging conditions with limited resources at Campbell Hospital in Calcutta, Brahmachari achieved a breakthrough in 1920. He synthesized urea stibamine, a compound that combined urea with para-amino phenyl stibnic acid. Urea stibamine significantly advanced the therapy for kala-azar. By 1925, the mortality rate had dropped to 10%, with a recovery rate of 95%. This new drug was not only a milestone in India but also proved effective in other countries, including Greece, France, and China [4,5,6]. Brahmachari’s dedication and innovative research saved numerous lives and left a lasting effect on the field of tropical medicine. By 1933, the Director of Public Health in Assam reported that the drug had been used to treat over 328,591 patients, saving countless lives and preventing the further spread of the disease. His work transformed a disease with a previously high fatality rate into one that could be effectively managed and treated, bringing hope and relief to millions affected by the disease. This groundbreaking achievement solidified his legacy as a pioneering physician and researcher in tropical medicine [2,4].

Research on dermal leishmanoid (Brahmachari) and other diseases

Brahmachari’s medicine, urea stibamine, was a groundbreaking treatment for kala-azar, saving countless lives. However, it caused adverse effects. In 1922, Brahmachari published “A new type of cutaneous Leishmaniasis,” where he documented skin eruptions in patients who recovered from kala-azar through intravenous antimony injections. Upon examining scrapings from these cutaneous nodules, he discovered that the skin eruptions were caused by cutaneous infection from the kala-azar parasite [7]. This significant finding was praised by JWD Megaw of the Indian Medical Services, who proposed naming the condition “Post-Antimonial Dermal Leishmaniasis” or “Brahmachari’s Dermal Leishmaniasis.” Brahmachari’s discovery was confirmed by Megaw, HW Acton, and other researchers. Notably, he was the first to observe that the disease could manifest even during the treatment for kala-azar. Together with his team, Brahmachari conducted extensive studies on the new disease, experimenting with treatments combining urea stibamine, neo-stibosan, and berberine. Later in his career, Brahmachari researched malaria, black fever, and the chemotherapy of quinoline and acridine compounds, aiming to develop effective medications. Although this research did not bring him the same level of recognition, it reflected his continued dedication to advancing medical science [4,7,8].

Remarkable achievements and legacy of Dr. U. N. Brahmachari

Throughout his illustrious career, Brahmachari held several prestigious positions and received numerous honors. He was awarded the Minto Medal in 1921 by the Calcutta School of Tropical Medicine, followed by the Kaiser-I-Hind Medal in 1924. He was also elected as a fellow of the Royal Society of Medicine in London in 1915, the Asiatic Society of Bengal in 1921, and the National Institute of Sciences of India (now INSA) in 1935. His leadership roles included serving as President of the Asiatic Society of Bengal, the Society of Biological Chemists, the Indian Chemical Society, and the Indian Association for the Cultivation of Science. Despite his immense contributions, Brahmachari faced challenges in gaining international recognition. His candidacy for the Fellowship of the Royal Society (FRS) and the Nobel Prize in Physiology or Medicine were hindered by circumstances such as the outbreak of World War II and the lack of international exposure and support. Notably, his FRS nomination, supported by eminent scientists such as Meghnad Saha, was never finalized due to wartime disruptions, and he passed away before the evaluation was completed. Similarly, his Nobel Prize nominations, though numerous, did not culminate in an award due to weak nominations and his limited global presence. Nonetheless, Brahmachari’s legacy endured through the honors he received from the British Empire, including the titles of Rai Bahadur and Knighthood, conferred upon him in recognition of his invaluable contributions to medical science. His pioneering work in the treatment of kala-azar and his dedication to medical research and education continue to inspire generations of scientists and healthcare professionals [1-3].

## Conclusions

Dr. Upendranath Brahmachari’s work in tropical medicine, particularly his development of urea stibamine for treating kala-azar, has had a profound and lasting impact on public health. His groundbreaking research not only saved numerous lives but also set a high standard for medical innovation. Despite challenges in gaining international recognition, his significant contributions were acknowledged through various honors and leadership roles. Brahmachari’s perseverance and dedication continue to serve as an inspiration for future generations in the medical field, highlighting the importance of innovative research and unwavering commitment to improving healthcare.
